# Annotations

**Published:** 1891-11-14

**Authors:** 


### ANNOTATIONS.

**"That poor worm, the doctor."** "That poor worm, the doctor, will sometimes turn," says the *British Medical Journal.* We are glad to hear it, and on such excellent authority. He has been turning, it seems, to some purpose in America. The medical practitioners of Stuttgart, in Arkansas, have been finding for a considerable time that their patients have such a high idea of medical philanthropy that they have allowed their medical men to live upon it almost entirely. In this country doctors' bills are often the last to be paid, but even that is better than the state of things in Arkansas, where they appear for the most part never to be paid at all. At last the doctors have actually combined, and formed themselves into a union; nay more, they have risen to the heroism of a strike. One of the rules of their union forbids any of its members to attend any sick person whose name is on a certain black-list of non-paying defaulters. The publication of this rule seems to have acted with quite explosive force on the inhabitants of the district. They have met in their hundreds to denounce those wicked practitioners who find it impossible to live and pay rent on their own philanthropy. The indignant citizens unanimously agreed to boycott every medical practitioner who should remain a member of the union for ten days after the date of their meeting. The conflict between the doctors and their gratuitous patients is, we believe, still in full swing. A man need not be a doctor in order to know to which side his sympathies should be given. It is really a question between the starving and the sick. Sickness is a bad thing, but starvation is a worse. If the sick are obliged to do without doctors, many of them will, no doubt, get better by the kindly help of nature; but if the doctors and their families cannot get money wherewith to pay for food, kindly nature will not step in to save them from the effects of starvation. Doctors in poor neighbourhoods occupy a very unenviable position, and sometimes even in rich neighbourhoods, too. It would be better both for them and the public if they would pluck up a little more courage, and hold together a little more in their own interests. So long as they remain isolated as they are at present they are entirely at the mercy of the least scrupulous section of the public. What that means many poor doctors know very well. It means all the difference between being able to live in modest comfort on the one hand, and slowly increasing debt and misery, often ending in disgrace and ruin, on the other. We are persuaded that appeals to the kindly feelings of the unscrupulous are mere waste of breath. Such persons are to be treated on purely business principles, and promptly compelled to render unto Cæsar the things that are his by the right of services which only Cæsar can render. The Arkansas doctors in their refusal to attend those who persistently decline to pay have set an excellent example to the whole of the medical world.

**An Undervalued Profession.** Is the chemist a professional man, or is he a mere shopkeeper? Undoubtedly he is a professional man, inasmuch as he cannot practice his calling without passing a somewhat severe examination for a State License. And what better is he for his State License? A great deal better as a public servant, but no better at all as a bread-winner for himself and his family; possibly sometimes worse. Every person who takes medicine seems to be anxious to cut down the chemist's prices. We do not find people grumbling at their butchers, their tailors, or their milliners; and yet where one chemist secures a modest competence, twenty butchers get immoderately rich. But the butcher does not require to pass any examination, and a mere trifle of capital is sufficient to set him up in business in a modest way. Sir James Sawyer the other day gave the public a much-deserved "wigging" on this chemists' question. Sir James as an experienced physician, is well entitled to speak on the subject. He told the public in set terms that it was by no means wise to be so stingy with its chemists. A great deal of the success of medical practice depends upon the trustworthiness of the drugs employed, and upon the experienced skill of their compounders. But a large section of the public are not willing to pay such prices as will secure first-class drugs; for the skill and experience of the chemist they are not willing to pay anything at all. Many people think a shilling a sufficient price to pay for a bottle of medicine of the ordinary six ounce size. No doubt it is possible to make up a mixture of Epsom salts or some other cheap concoction at a shilling the six ounces. But an average charge of two shillings for ordinary six ounce mixtures is very moderate. It should be remembered that chemists as a class are well educated and specially trained professional men. It is of the utmost importance that they should be men of culture and character. But the public is going the right way to disestablish men of culture and character, and to force into their places men who have neither culture nor character. We do not appeal to intelligent readers on behalf of a class of ill-used specialists *ad misericordiam,* but we do ask the public to use sufficient sense for the protection of its own interests, and its interests emphatically demand that those who compound its drugs shall be honest, capable, and fairly paid men.

**An Important Hospital Experiment.** Some time ago the Great-Northern Central Hospital, which during the last few years has ranked with the important general hospitals of London, had reason to believe that its out-patient department was made use of to a considerable extent by persons who could afford to pay private medical attendance. The managers resolved that they would make a vigorous effort to put an end to a state of things which was at once discreditable to the well-to-do out patients, and injurious both to the funds of the hospital and to those really poor persons who have no resource in sickness but charitable medical relief. Accordingly they appointed a fully qualified and competent casualty medical officer. The duties of this officer were to deal personally with the slighter cases; to discover, if possible, any persons who, being able to pay for private medical attendance had no right to hospital treatment; and also to obtain from applicants who might seem doubtful, satisfactory proof that they were really proper cases for charitable medical relief. The report issued deals with 6,540 new applicants. Of these 6,540 cases, nearly a third, or to be precise, 2,259 were found to be suffering from ailments of quite a trivial character, and were summarily dealt with by the casualty officer. In other words, a provident dispensary, and not a general hospital, was the proper place for them. Forty-eight persons, who openly stated that they came on the real or pretended supposition that they would receive better treatment at the out-patient department of the hospital than they could obtain at the hands of private practitioners, and who did not attempt to deny their ability to pay for the best medical treatment, were refused admission altogether. Those forty-eight persons, if dissatisfied with their own medical men; and desirous of taking the advice of hospital physicians, should have gone, not to the hospital, but to the consulting-rooms of those hospital physicians and paid their guinea or their two-guinea fees. A couple of guineas is not a very alarming fee, even for persons of moderate means. If such persons cannot afford the two guineas, they can get excellent advice in any part of London for five shillings. Eleven of these prosperous-looking applicants for hospital relief declined altogether to give particulars about themselves, and preferred to go away. Who were the eleven? Possibly some of them were present in the borrowed dresses of their own cooks. As many as 454 cases, or about one in twelve of the total number applying, seemed to be persons whose circumstances demanded investigation, and it was found on inquiry that no fewer than 209 of them were in such prosperous circumstances as to be disqualified for hospital relief. Now, putting together these four classes of trivial cases, cases of confessed competence, cases declining investigation, and cases proved by investigation to be unsuitable, we have in a total of 6,540 no fewer than 2,627 individuals who had no proper claim to hospital relief. If this represents the proportion of unsuitable cases that apply to all the metropolitan hospitals—and there is no reason to doubt that it does—then it is plain that more than a quarter of the medical relief given in London is unnecessary and is an injury rather than an advantage to those who receive it. We repeat, for the twentieth time, that this state of things must not be allowed to continue. It is a public scandal. The hospitals would gladly put an end to it. They must put an end to it by combining and carrying out some common plan for their own defence. It is a subscribers' question, and if the subscribers insist upon it the scandal will soon be abolished.

